# Opening the OPK Assay Gatekeeper: Harnessing Multi-Modal Protection by Pneumococcal Vaccines

**DOI:** 10.3390/pathogens8040203

**Published:** 2019-10-23

**Authors:** Ashleigh N. Riegler, Beth Mann, Carlos J. Orihuela, Elaine Tuomanen

**Affiliations:** 1 Dept. of Microbiology, University of Alabama at Birmingham, Birmingham, AL 35294, USA; ariegler@uab.edu (A.N.R.); corihuel@uab.edu (C.J.O.); 2 Dept. of Infectious Diseases, St. Jude Children’s Research Hospital, Memphis, TN 38105, USA; beth.mann@stjude.org

**Keywords:** opsonophagocytosis killing assay, vaccine-induced protection, non-opsonic antibody

## Abstract

Pneumococcal vaccine development is driven by the achievement of high activity in a single gatekeeper assay: the bacterial opsonophagocytic killing (OPK) assay. New evidence challenges the dogma that anti-capsular antibodies have only a single function that predicts success. The emerging concept of multi-modal protection presents an array of questions that are fundamental to adopting a new vaccine design process. If antibodies have hidden non-opsonic functions that are protective, should these be optimized for better vaccines? What would protein antigens add to protective activity? Are cellular immune functions additive to antibodies for success? Do different organs benefit from different modes of protection? Can vaccine activities beyond OPK protect the immunocompromised host? This commentary raises these issues at a time when capsule-only OPK assay-based vaccines are increasingly seen as a limiting strategy.

## 1. Introduction

The development of vaccines has historically placed great emphasis on three gold standard functional assays of antibody activity: the opsonophagocytic killing (OPK) and serum bactericidal assays for bacteria and the neutralization assay for viruses. The pneumococcal vaccine is no exception, and design has principally included only capsular antigens, which are held only to the OPK assay standard. This approach presents a narrow vision of both immunogen and antibody function. A growing body of evidence indicates that this single-minded approach is passé, and that not only can anti-polysaccharide antibodies do much more than enable OPK, but new protein antigens can also be protective even in the absence of neutrophils. This commentary addresses the question, “In an era recognizing a need for broader anti-pneumococcal vaccines that protect against old and newly recognized aspects of disease, should measuring vaccine efficacy be modified by adding other assays of functions of both capsules and proteins as immunogens?” We posit that capsular polysaccharide and protein immunogens have much broader bioactivities in terms of both quantity and quality of immune functions, which can be targeted and harnessed as better vaccines are developed. 

## 2. Discussion

### 2.1. Multi-Modal Protection: Anti-Capsular Antibodies Are Not Just Active in OPK Assay

In a striking demonstration of the limitations of a single assay approach to predicting vaccine efficacy, Doyle and Pirofsky described two mouse monoclonal antibodies, 7A9 and 1E2, raised against a pneumococcal capsular polysaccharide [1]. 7A9 killed pneumococci in the OPK assay, but 1E2 did not. However, both antibodies protected equally well against lethal challenge in mice. This result clearly challenged dogma, and shouted loudly that it is not true that a “good” polysaccharide-based vaccine must be active in an OPK assay. If antibody function is complex, why focus on measuring only part of it? A clear clinical impact of non-opsonic antibody activities can be seen in the setting of neutropenia, such as in the growing population of patients undergoing cancer chemotherapy. Would the gaps in capsular vaccine protection in non-sepsis models be filled by these alternative activities?

In 1937, Harry Eagle described two dissociable activities of anti-pneumococcal horse serum: aggregation of bacteria vs. protection in animal models of sepsis [2]. As early efforts in vaccine design appropriately focused on preventing sepsis, which was reflected in OPK activity, agglutinating activity was rarely studied. Since 2000, the Pirofsky lab has examined the bioactivities of several protective, non-opsonic anti-capsular antibodies and showed that protection was correlated with the ability to agglutinate bacteria [3]. Agglutination, a feature of the state of natural transformation, modulates quorum sensing and specifically increases bacterial death by fratricide [4]. In 2017, it was recognized that anti-capsular antibodies that agglutinate bacteria interfere with pathogen shedding from the respiratory tract and subsequent transmission [5]. Mitsi et al. further demonstrated that the agglutinating activity of antibodies generated by vaccination with capsule were critical to protection against acquisition of carriage in a human challenge model [6]. An interesting twist to this emerging story is the recent demonstration that the influenza virus can hitchhike on aggregated pneumococci, and thus strongly enhance transmission of both pathogens [7]. It stands to reason that agglutinating activity could be a major predictor of the ability of antibodies to protect from spreading disease, and that distinguishing antibodies that protect in sepsis vs. colonization/transmission may require very different assays.

What else do capsules do that can be harnessed for vaccines? Although not tested as yet, a short list of possible modes of protection by anti-capsular antibodies can be generated by focusing on the multiple modes of bacterial clearance (Table 1). Antibodies could enhance the ability of neutrophil extracellular traps (NETS) to capture pneumococci [8,9]. Antibodies could counteract the ability of capsule to blunt the release of cytokines such as IL-6 and IL-8 from epithelial cells during the acute phase response [10]. Some non-opsonic, protective, anti-capsular monoclonal antibodies have been shown to decrease IL-8 secretion from leukocytes [11]. These examples suggest that protective activity in vivo not only can be, but most probably always is multi-modal, and can be recruited to expand vaccine efficacy even for the single polysaccharide capsule family of antigens.

### 2.2. Multi-Modal Protection: Protein Functions Enter the Vaccine Efficacy Assay Repertoire

Introduction of protein antigens to the vaccine design discussion opens up a wide array of bioactivities that could be folded into standalone or existing vaccines to potentially improve prevention of disease (Table 1). Proteins with conserved sequences would build serotype-independent protection and interrupt more pathophysiological steps that would be expected to improve vaccine impact, than just OPK, especially outside of the bloodstream. It is well accepted that antibodies against bacterial surface proteins interfere with bacteria–host cell interactions, particularly on mucosal surfaces, and thus interrupt the progression of disease from site to site. This reasoning would support developing these antigens to improve vaccine-induced activity, especially against pneumonia, otitis media, and nasopharyngeal carriage.

A major hurdle for protein-based vaccines is the definition of an assay related to the protein’s function that would correlate to protection and predict improved vaccine efficacy. Particularly attractive would be activities in sites in which capsule-based vaccines show deficient protection: pneumonia, otitis media, or nasopharyngeal carriage. Many in vitro and in vivo assays have shown the bioactivities of many pneumococcal proteins, but adding them into vaccines requires assays that accurately correlate a functional assay with protection, often independent of prevention of sepsis. The difficulty of implementing this requirement was illustrated by a recent Phase II clinical trial of a combination of 10 polysaccharides with the histidine triad protein PhtD and a pneumolysin toxoid [12]. PhtD is believed to participate in zinc homeostasis in the nasopharynx, and the toxin pneumolysin causes damage to respiratory epithelial cells during pneumonia; both vaccine antigens decreased nasopharyngeal carriage in preclinical models [13,14]. However, the only functional assay developed to test sera after this combined vaccine was not directed to mucosal activity, but rather focused on assessment of complement-mediated killing by leukocytes [15]. This is essentially an OPK assay, which is not expected to be highly relevant to nasopharyngeal carriage. One trial in Gambian infants reported that the proteins produced high titer antibodies but failed to decrease nasopharyngeal carriage of any pneumococci (serotypes within or outside those in the vaccine) [12]. The key question remains: Did this failure arise because the antigens failed to generate antibodies (quality and quantity) directed to mucosal events in disease? Or were toxin neutralizing antibodies and anti-PhtD antibodies doing their jobs, but this approach was truly not effective in prevention? In the first case, the antigens have not really been tested; in the other, they are likely not useful vaccines. If they are not good vaccines, then this example of divergence in vaccine efficacy in preclinical animal models versus humans highlights the limits of animal models, and supports the continuation and expansion of human carriage trials for new vaccine candidates.

Noncapsular antigens can clearly protect against disease. For instance, virtually all respiratory bacterial pathogens display phosphorylcholine on their surfaces, and this decoration has been shown to enable several pathogens to interact with host cells via the platelet activating factor receptor [16,17]. C-reactive protein, a primordial member of the acute phase response, effectively counteracts this shared mechanism of invasion, illustrating non-capsule-based protection [18]. Similarly, shared motifs for the binding of bacteria to laminin receptors indicate that antibodies to a single bacterial protein can be broadly cross-protective against pathogens that use the same invasive strategy. This has been demonstrated using immunization with the pneumococcal laminin receptor binding protein, choline binding protein A (CbpA/PspC), resulting in protection against pneumococcal, *Haemophilus*, and meningococcal diseases [19,20,21,22]. Should this antigen strategy be developed into a vaccine, it is expected that the serum would need to be tested not just for high titer antibodies, but also for a high level of antibodies active in a functional assay reflecting CbpA binding to laminin receptor in vitro. 

The process of shepherding protein-based vaccines through clinical trials will require reworking or replacing the OPK assay with functional assays for many other modes of defense. Revamping the OPK assay would be appropriate for testing whether blocking bacterial complement-binding proteins protects against activation of the complement cascade in phagocytosis. Complement-binding proteins of pneumococcus include the surface adhesin CbpA/PspC, which has been demonstrated to recruit Factor H from serum to inhibit amplification of C3b activation. Antibodies to CbpA/PspC block the interaction with Factor H, and thus allow complement fixation to continue [23,24,25].

For most activities of proteins in pathogenesis, new assays will need to be deployed in the clinic. Similarly to neutralization standards for viral vaccines, pneumolysin toxoid vaccines will need to be measured not only for the ability to neutralize pneumolysin-induced hemolysis, but also for prevention of the oligomerization required for pore formation [26]. Matrix-binding proteins, such as PavA, PavB, PfbA, RrgA, and CbpA/PspC, contribute to invasion of tissues, and inhibition of the progression of invasion by antibodies can be quantified by binding to fibronectin, vitronectin, and other matrix components [27,28,29,30]. A more complex approach is needed to ascertain effective blockade of the biofilm formation known to participate in nasopharyngeal colonization and otitis media. Many of the steps in biofilm formation cross over into bacterial aggregation (discussed above) [31,32]. It is clear that the physiology of biofilms and virulence extends to changes in bacterial behavior such as competence and fratricide [33,34,35,36]. Many proteins and signaling molecules, such as PsrP, GlpO, BriC, CbpD, LytC, and CbpF, can be targeted to interrupt the participation of these processes in biofilms and colonization. Others approaches involve distinguishing the features of bacteria shed from biofilms that then move on to produce disease in other organs or enhance transmission [7,37].

### 2.3. Multi-Modal Protection: Prevention of Disseminated Disease and Toxicity

Approximately 30% of adults hospitalized for pneumococcal pneumonia go on to develop bacteremia [38]. Critically, pneumococci in the bloodstream can disseminate and directly contribute to secondary organ damage independent of sepsis; some stark examples include neuronal damage in the central nervous system as result of meningitis and cardiomyocyte death following bacterial translocation into the myocardium [39,40]. Clinical epidemiological studies indicate that survivors of invasive pneumococcal disease are at greater risk for development of frailty, loss of independence, and a shortened lifespan [41]. Thus, an antibody that neutralizes the bacterial factors responsible for pneumococcal invasiveness and/or its toxicity would offer vital protection in a manner that is not covered by serotype-specific OPK.

Antibodies against a variety of pneumococcal adhesins have been demonstrated to reduce the ability to adhere to host cells and thus attenuate disease severity in murine models. Perhaps the best characterized, antibodies specific for CbpA/PspC have been shown to inhibit the ability of *Streptococcus pneumoniae* to bind polymeric immunoglobulin receptor, its ligand on mucosal epithelial cells, and 37 kDa laminin, its ligand on vascular endothelial cells [19,42]. As interactions with these receptors are involved in the uptake of pneumococci into endosomes and their translocation to the basolateral surface, immunization with CbpA inhibits the development of bacteremia, meningitis, and the formation of cardiac microlesions in animal models [19,43]. Targeting this organ-specific toxicity could be a significant upgrade to even existing capsule-based vaccines.

Pneumolysin is a pore-forming toxin produced by all clinical strains of *S. pneumoniae* [44]. It is thought to be responsible for much of the inflammation observed during pneumonia and the organ damage observed during disseminated infections. Briefly, pneumolysin has been directly implicated in hearing loss during otitis media, lung injury during pneumonia, neuron death during meningitis, and the killing of cardiomyocytes (Table 2) [14]. Being a pore-forming toxin, pneumolysin disrupts ion regulation. At low concentrations, this disrupts normal cell function, such as cardiomyocyte contraction and immune cell function, whereas higher concentrations kill host cells outright by various means, depending on exposure level and cell type [45,46,47]. Pneumolysin is not secreted by the bacteria, nor is it found on the bacterial surface at high concentrations. It is instead released from the bacteria as result of its death [48]. Thus, a neutralizing antibody against this toxin would have limited efficacy in an OPK assay. One emerging understanding is that this toxin plays a key role in instances where the pneumococcus is replicating in a fixed region within tissues. Along such lines, antibodies against pneumolysin have been demonstrated to protect against cell death in vitro and diminish the tissue damage that occurs during severe pneumococcal disease [49]. Pneumolysin also has other activities, such as activation of the classical complement cascade and binding to Toll-like receptor 4 to trigger inflammation [50,51]. Presumably, antibodies that block these interactions would also reduce the inflammatory damage that occurs during infection [51]. Importantly, the negative consequences of disseminated *S. pneumoniae* infection and pneumolysin toxicity are most likely already ongoing at the time of initial antimicrobial therapy in a patient care setting. Moreover, cell-wall-acting antimicrobials may worsen tissue injury, given the bolus of pneumolysin that would be released with bacterial death. Thus, immunization to protect against invasive disease caused by all serotypes of *S. pneumoniae* and to neutralize pneumolysin toxicity would be the protective method of choice.

Critically, molecular genetics allow the construction of hybrid molecules that contain both adhesin and toxin neutralizing epitopes. Multivalent pneumococcal protein vaccines that are composed of pneumolysin and fragments of key adhesins have been tested and shown to elicit strong and broad protection [69]. Such antibodies are thought to act by limiting the ability of pneumococci to attach to host cells and translocate, and, in addition, by neutralizing pneumolysin toxicity. Again, these are aspects of protection that would not be evident in a standard OPK assay. 

### 2.4. nonIgG Immunological Correlates and Cellular Responses

For opportunistic mucosal pathogens like *S. pneumoniae*, secretory immunoglobulins play a pivotal role in pathogen control before the development of disease. While *S. pneumoniae* expresses an IgA protease that limits the impact of anti-pneumococcal IgA, previous work has shown that secretory IgA (sIgA), the polymeric form of the antibody, is capable of contributing to pathogen control through agglutination, as well as by facilitation of complement-mediated killing [70,71,72]. These systemic and site-specific IgA responses are not currently measured by the standard OPK assay and, we suggest, would be valuable measures of vaccine efficacy at the mucosal sites. Additional quantitation, such as a selective ELISA or agglutination efficacy of immunoglobulins from nasal and oral secretions, could provide valuable information on the site-specific responses elicited by the vaccine.

Another neglected aspect of potential vaccine-induced immunity against pathogens is the production of non-humoral cellular immunity at the interface of innate and adaptive responses. Activation of receptors on the surfaces of epithelial and immune cells at mucosal sites by pneumococcal proteins, such as TLR 2 interactions with cell wall–protein complexes or TLR 4 activation by pneumolysin, alter the inflammatory transcriptome of interacting cells, directly influencing the endocytosis of the pathogen [51,52,73]. The production of cytokines such as IL-1, IL-8, and thymic stromal lymphoprotein by epithelial cells when they encounter a foreign antigen has profound effects on the activation states of antigen-presenting cells such as dendritic cells, as well as the development of an antigen-specific T-cell repertoire [74,75,76]. Notably, it is now appreciated that CD4^+^ T cells specific to various pneumococcal protein antigens are vital in the initiation of a robust response within the respiratory tract and at other mucosal sites (reviewed by Reference [77]). CD4^+^ cellular responses to pneumococcal protein antigens have been shown in mice and humans to increase bacterial clearance from the airway through IL-17 mediated recruitment of macrophages and neutrophils [78,79] as well as by IL-10 and interferon-regulated mucosal antibody production [80,81]. The necessity for this CD4^+^, Th17-like response is supported by a decrease in incidence and severity of pneumococcal infections with age, independent of serum levels of anti-pneumococcal antibody [82], as well as the increased susceptibility to pulmonary infection in humans with deficient IL-17 signaling [83,84]. The possible side effects of the pro-inflammatory activity of Th17 cells, particularly on second challenge, remains a potential issue for vaccine safety. Additionally, mice deficient in B-cell antibody responses (uMT mice) clear primary pneumococcal colonization over time, while mice deficient in CD4^+^ T cells are susceptible to colonization and lack protection from re-colonization [73,85,86]. Re-stimulation of antigen-specific cells could be used to detect such Th17-like responses. While analysis of human B- and T-cell responses to vaccination are difficult to assess, requiring tissue collection and antigen activation assays ex vivo, in these instances, the above animal models provide valuable insight into the immune processes during infection and in response to vaccination which are not mediated by antibodies, and should be considered vital in the initial assessment of potential vaccine candidates. 

## 3. Conclusion

As we begin to recognize the clinical limitations of current pneumococcal vaccines and consider approaches towards the rational design of an improved vaccine against *S. pneumoniae,* we suggest that the current standard of evaluating vaccine efficacy must also evolve beyond the OPK assay. For *S. pneumoniae*, a tremendous amount of data has shown that control and eradication of the bacteria during infection is reliant on many mechanisms, not simply the generation of opsonizing antibody. Antibodies that aggregate bacteria, impair bacterial–host cell binding, promote complement- mediated killing, disrupt bacterial signaling, enhance killing by NETs, and modulate immune cell responses are all potent mechanisms of potential vaccine-induced pathogen control which the OPK assay ignores. Knowing how each of these defense mechanisms applies to the many organs damaged by pneumococcal infection will also provide expanded avenues for designing better multi-modal vaccines.

## Figures and Tables

**Table 1:** Non-opsonic activities of antibodies that could improve prevention by vaccines.

Anti-Capsule Activity	Anti-Protein Activity
Increase agglutination	Increase agglutination
Enhance NETS	Neutralize toxin
Increase cytokines	Block adherence to cells, matrix Block biofilm components, competence Enhance fratricide Block metabolism, e.g. uptake of metals, sugars

**Table 2:** Tissue damage attributed to pneumolysin during *Streptococcus pneumoniae* infection.

Affected Site	Targeted cell	Consequence	Ref.
Nasopharynx	Mucosal epithelial cells	Epithelial cell sloughing	[52,53]
Middle ear	Cochlear hair cell	Hearing loss	[54]
Lower respiratory tract	Bronchial epithelial cells Alveolar epithelial cells	Inhibited mucociliary clearance, lung consolidation, pulmonary fibrosis	[46,55,56,57]
Central nervous system	Neurons, brain endothelial cells, ependymal cells	Cognitive impairment	[58,59,60,61,62,63]
Heart	Cardiomyocytes	Impaired contractility, cardiac remodeling	[43,45]
Immune system	Macrophages, neutrophils, dendritic cells	Reduced clearance, dampened immune signaling, enhanced pneumococcal survival	[64,65,66,67,68]
